# Changes in vaginal gene expression and anogenital distance during gilt reproductive development

**DOI:** 10.1590/1984-3143-AR2024-0056

**Published:** 2024-12-20

**Authors:** Shannon L. Dierking, Jodi M. Morton, Jeffrey A. Clapper, Michael G. Gonda, Juan C. Pinilla, C. L. Levesque

**Affiliations:** 1 CSA Animal Nutrition, Dayton, OH, U.S.A; 2 Department of Agricultural Sciences, Southern University and A&M College, Baton Rouge, LA, U.S.A; 3 Department of Animal Science, South Dakota State University, Brookings, SD, U.S.A; 4 Topigs Norsvin, USA, Burnsville, MN, U.S.A

**Keywords:** estrus, puberty, reproduction, sow, and swine

## Abstract

Early puberty is associated with improved long-term reproductive performance. Predicting who will achieve early puberty is limited to intensive, invasive serial blood collections for measurement of reproductive hormones. The vaginal genome during pubertal development has potential as biomarkers of early estrus in the pre-pubertal period. Pre-pubertal gilts (n =13) were followed from d74 ± 3 of age until first estrus or d214 ± 1 of age. Blood, vaginal epithelia, and anogenital distance were collected at five timepoints during reproductive development (d74, d104, d130, d160 and first estrus or end of trial). Total RNA was isolated from vaginal epithelia and relative gene expression of two toll-like receptors (TLR-4 and TLR-5), tacykinin precursor-3 (TAC-3), insulin-like growth factor-1 (IGF-1), and estrogen receptor (ERα)-alpha was quantified by real time RT-PCR, relative to expression of RPLP0. Four gilts exhibited estrus early (< d184), 3 were average (d194 to 195), 3 were late (d203 to 213), and 3 were deemed anestrus. Comparison of expression of each gene relative to d70 was performed using the PCR package in RStudio (version 1.2.5025) and Fisher’s exact t-test for TLR-4, TLR-5 and TAC-3, and ANOVA for ER-alpha and IGF-1. Correlation analysis examined the relationship between anogenital distance and age at first estrus. A single blood draw for serum progesterone was obtained 8 days after recorded first estrus or end of trial; the presence of serum progesterone supports the visual identification of standing estrus. Expression of IGF-1 and TAC-3 were up-regulated 9- and 7-fold, respectively at d160 (*P* < 0.05). Expression of ERα tended to be upregulated 3-fold at d104 (*P* = 0.08) and expression of TLR-4 and TLR-5 was not detected until first estrus. Anogenital distance was positively correlated to the first estrus. These transcripts associated with reproduction warrant further investigation into use as biomarkers to detect early estrus.

## Introduction

The best-known predictor of reproductive success in gilts is age at puberty (Rosendo et al., 2007; Kuehn et al., 2009). However, the ability to predict which females will achieve early estrus is currently limited to intensive, invasive collections of serial blood samples for measurement of reproductive hormones (Sasaki and Koketsu, 2008), which is not practical for on-farm use. Physical characteristics such as anogenital distance and vulva width are proposed as a practical tool for prediction of age at puberty in the prepubertal period because changes in size have been associated with reproductive development in gilts (Romoser, et al. 2020; PIC, 2017; Seyfang et al., 2018) and can be measured without frequent blood sampling of reproductive hormones. With advancements in the field of ‘omics’, advanced molecular techniques hold promise to develop predictive markers and development of precision breeding technologies. Recent work identified associations between host vaginal lipidome, and bacterial composition and gilt fertility, immune stimulation, breeding strategy, vaccination status, and pregnancy (Sanglard et al., 2020; Luque et al., 2021; Mills et al., 2021; Alves et al., 2022). However, these methods do not specifically focus on periods during reproductive development in the pre-pubertal phase as possible predictors of age at puberty. Therefore, the objective of this study was to measure anogenital distance and characterize changes in the vaginal epithelium of gilts at key time periods during reproductive development.

## Methods

### Animal care

A total of 13 pre-pubertal gilts (PIC 1050; 34 ± 8 kg; d74 ± 3 days of age) were housed at South Dakota State University Swine Education and Research Facility. Gilts were provided a common diet in 5 phases with ad libitum feed intake and nutrient specifications required for growth (Swine Nutrition Guide, 2010; Table 1) as well as ad libitum water access via 1 cup waterer per pen or stall. Females were housed in groups of 10 until approximately d150 of age, when they were then transitioned to individual stalls. Diets were formulated to 1507 kcal metabolizable energy (ME)/kg, 1.08% digestible lysine and 1.12 Ca/P ratio (phase 5), 1513 kcal ME/kg, 0.94% digestible lysine and 1.12 Ca/P ratio (phase 6), 1517 kcal ME/kg, 0.84% digestible lysine and 1.11 Ca/P ratio (phase 7), 1519 kcal ME/kg, 0.75% digestible lysine and 1.12 Ca/P ratio (phase 8) and 1490 kcal ME/kg, 0.55% digestible lysine, and 1.23 Ca/P ratio (gestation diet). Animal procedures were reviewed and approved by the South Dakota State University Institutional Animal Care and Use Committee (approval number 19-006A).

**Table 1:** List of primers by gene, sequence, and amplicon size (bp).

**Gene**	**Primer**	**Amplicon size, bp**
RPLP0^1^	Forward: 5’ – CTGAGTGATGTGCAGCTGATTA – 3’ Reverse: 5’ -CCCGTGTGTACCCATTGAT – 3’	137
ER-α2	Forward: 5’ – GAATGTTGAAGCACAAGCGCCAGA – 3’ Reverse: 5’ – ACCGGGCTGTTCTTCTTAGTGTGT – 3’	91
IGF-13	Forward: 5’ – AGAACTGCACGGTGATCGAG – 3’ Reverse: 5’ – AGATGACCAGGGCGTAGTTG -3’	146
TAC-34	Forward: 5’ – GACTTCTTTGTGGGTCTTATGG – 3’ Reverse: 5’ – GCAGTTTCTACAGACGGTGG – 3’	122
TLR-45	Forward: 5’ – TCATCCAGGAAGGTTTCCAC – 3’ Reverse: 5’ – TGTCCTCCCASCTCCAGGTAG – 3’	186
TLR-56	Forward: 5’- GGTCCCTGCCTCAGTATCAA – 3’ Reverse: 5’ – TGTTGAGAAACCAGCTGACG – 3’	187

^1^: porcine RPLP0; ^2^porcine estrogen receptor-alpha; ^3^porcine insulin-like growth factor 1; ^4^porcine tachykinin-receptor 3; ^5^porcine toll-like receptor 4; ^6^porcine toll-like receptor 5.

### Sample collection

**Figure 1:**
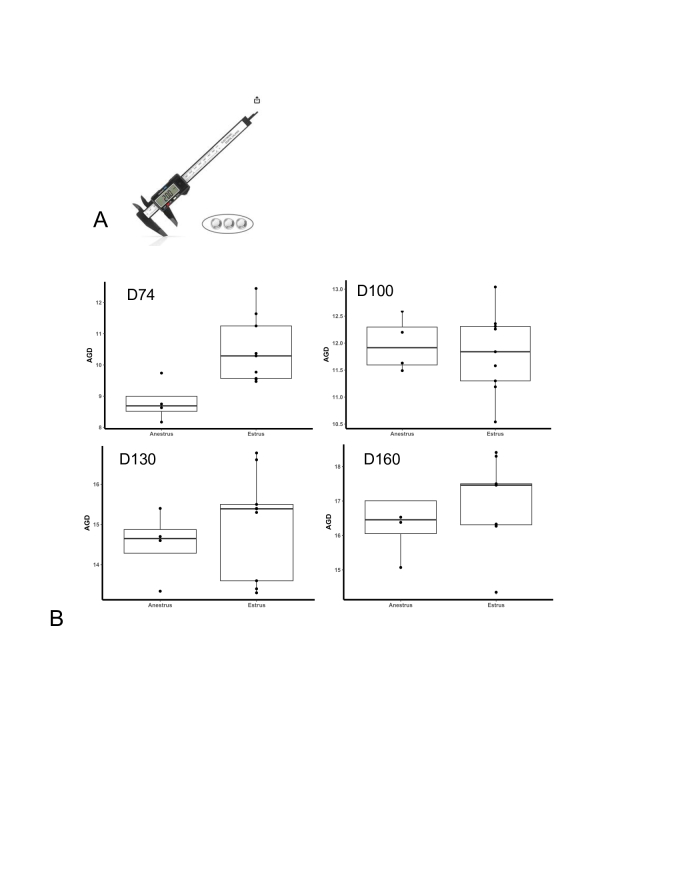
Digital caliper used to measure anogenital distance (A) and anogenital distance at d74, d104, d130, and d160 of age between gilts that achieved estrus (Estrus) by day 214 of age and those that did not (Anestrus) (B).

Vaginal swabs and blood were collected and anogenital distance and body weight measured at 5 timepoints during predicted reproductive development: d74 (prior to folliculogenesis), d104 [mid-folliculogenesis which occurs in swine between d80-120 of age (Anderson, 1980)], d130 (post-folliculogenesis), d160 (start of daily boar exposure) and a final collection at either first estrus or end of trial (d214), whichever came first. Vaginal swabs were collected using a Cervex-Brush cervical cell sampler (Fisherbrand #1437256, Fisher Scientific, Waltham, MA) placed approximately 4 cm into the vagina and 3 partial hand rotations using slight pressure to collect tissue from the dorsal portion and sides to avoid the suburethral diverticulum. The cytology brush was placed in a cryovial containing TRIzol reagent (Invitrogen, Waltham, MA) and swished for 30 seconds; the reagent was flash frozen in liquid nitrogen and stored at -80^o^C prior to RNA extraction. Blood sample (3 mL) was collected via jugular venipuncture [20 g x 1.5-inch needle; serum vacutainer (BD Vacutainer, Franklin Lakes, NJ). Gilts were held in the weigh scale for anogenital distance measurements to aid in keeping the females calm to reduce clenching of the vulva during measurements. For each gilt, anogenital distance was measured using digital calipers (Figure 1a) and recorded in triplicate by the same technician at each time point to maintain consistency. The anogenital distance was considered the distance from the top of the vulva opening to the middle of the rectum. At d160, following sample and data collection, boar exposure (defined as 15 min of fenceline contact) was started and continued daily from 08:00 h. Two mature boars, housed separately from the gilts, alternated daily. Standing estrus was detected by back pressure and human identification of estrus behavior (i.e., ‘locked’ stance, ears erect, grunting, swollen, pink vulva). Once standing estrus was detected a vaginal swab was collected and anogenital distance measurement recorded. A single blood sample via jugular venipuncture was collected at d8 following first estrus to confirm ovulation occurred based on serum progesterone concentrations of 1 ng/mL or greater (Guthrie, 2005).

Total RNA was isolated from vaginal epithelium (Morton, 2018) for measurement of relative expression of genes involved in the immune response [toll-like receptor-4 and 5 (TLR-4 and TLR-5) and tachykinin precursor-3 (TAC-3)], whole body growth [insulin-like growth factor-1 (IGF-1)], and in the development of the female phenotype [estrogen receptor-α (ER- α)]. Expression of TLR-4 and TLR-5 were not detected at d 74; therefore, the expression of TLR-4 and TLR-5 was calculated relative to d104. It should be noted that TLR-4 and -5 were lowly detected, in samples at d104, d130 and d160 (n = 3/13, 2/13, and 2/13 samples). Real-time semi-quantitative PCR was used to measure the quantity of vaginal ER-α, IGF-1, TAC-3, TLR-4 and TLR-5 mRNA relative to the amount of porcine RPLP0 mRNA in each sample (Table 1). Measurements of the relative quantity of the cDNA of interest was carried out using RT2 Real-Time™ SYBR Green/ROX PCR Master Mix (SuperArray Bioscience Corp., Foster City, CA). Twenty microliter reactions were measured using the Stratagene MX3005P quantitative real-time PCR instrument (Agilent Technologies, Foster City, CA). Thermal cycling conditions recommended by the manufacturer (40 cycles of 30 sec at 95ºC, 1 min at 55ºC, and 1 min at 72ºC) were used for all genes. The concentrations of forward and reverse primers used for the genes of interest and RPLP0 were 300 nM. A linear response was obtained when these concentrations of primer pairs were used with increasing amounts of cDNA. Dissociation curve analysis was performed after each real-time PCR run to confirm that a single amplicon was present. Blood samples were left at room temperature and allowed to clot before centrifugation at 2,500 rpm for 15 minutes at 22^o^C. Serum was collected and stored at -20 ^o^C until further analysis.

## Statistical analysis

A t-test was performed in RStudio (v10.0.2) to compare AGD measurements at days 74, 104, 130, and 160 between females identified as achieving estrus by day 214 and those that did not (anestrus). Fold-change of each gene relative to expression at d74 was determined using the PCR package in Rstudio (version 1.2.5025). Analysis of gene expression utilized Fisher’s exact t-test for genes TLR-4, TLR-5 and TAC-3, and ANOVA for genes ER-alpha and IGF-1. Two different analyses were utilized due to low expression for TLR-4, TLR-5 and TAC-3, and a reduced number of timepoints where expression of these genes were detected.

## Results

### Anogenital distance 

There was a difference in AGD at d74 between estrus and anestrus females (*P* = 0.01), with estrus-grouped females having a longer AGD compared to anestrus (Figure 1b). There were no differences detected between the groups at days 104, 130, or 160.

### Hormone concentrations

 Serum concentration of progesterone was not detectable (i.e. <1 ng/mL) at any timepoint prior to d160. Five of the 13 gilts had not expressed behavioral signs of estrus by the end of trial (d214 of age ± 1). In 3 of these females, serum progesterone concentration were < 1 ng/mL indicating lack of estrus. In two gilts, serum progesterone concentrations at either d160 (10.7 ng/mL) or d215 (14.9 ng/mL) indicated that ovulation had occurred despite the lack of physical estrus behaviors and indicated an undetected or ‘silent’ heat. Females that achieved a detected estrus had serum progesterone concentration of 21.033 ± 5.10 ng/mL.

### Gene expression

**Figure 2:**
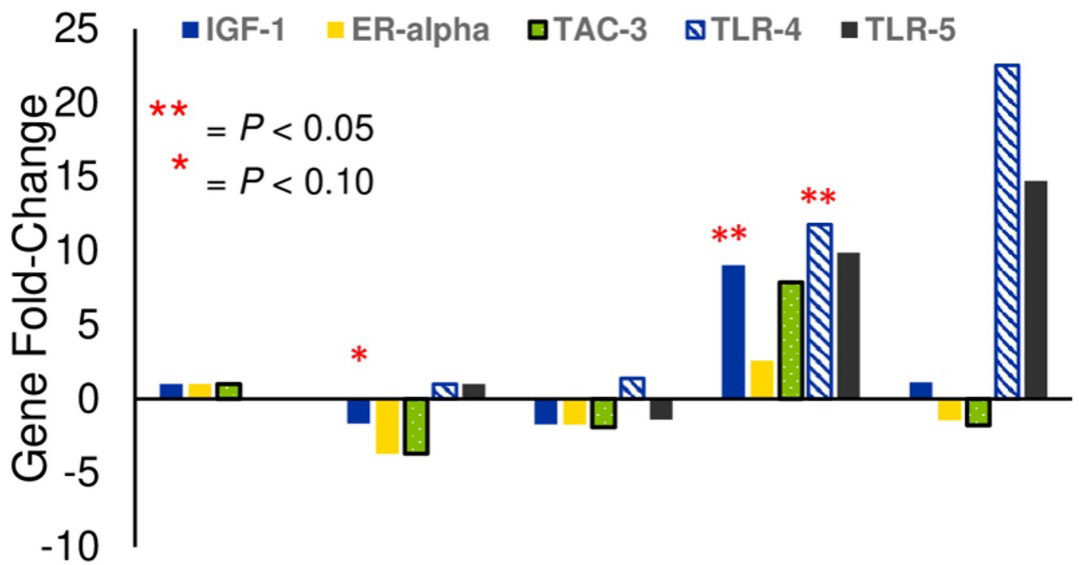
Changes in gilt vaginal gene expression at key time points during reproductive development. D74 represents the early prepubertal period, d104 represents mid-folliculogenesis, d130 represents post-folliculogenesis, d160 represents start of boar exposure, d214 or S.E. (first standing estrus) represents end of trial. Relative expression of IGF-1, ER-alpha, and TAC-3 at each time point were compared to expression at d74. Due to low expression at d74, relative expression of TLR-4 and TLR-5 at d130, 160, and trial end were compared to expression at d104.

Relative expression of ER-α in vaginal swabs tended to be upregulated 3-fold at d104 (*P* = 0.08; Figure 2). At d160, there was a 9-fold increase in relative IGF-1 expression (*P* = 0.02), a 2-fold increase (*P* = 0.08) in ER-α expression, and a 7-fold increase (*P* = 0.04) in TAC-3 expression. As described in the materials and methods, expressions of TLR-4 and TLR-5 were not detected or were lowly detected. In part due to low expression, no differences were detected in TLR-4 and TLR-5 expression over time; however, at first estrus or end of trial, expression was readily detected in 8/13 samples, with expression of TLR-4 and TLR-5 being upregulated 22-fold and 14-fold, respectively.

## Discussion

Simple, easy to apply tools to estimate age at first estrus in the prepubertal stage has potential to address on-farm labor concerns and improve overall sow farm productivity by allowing focus on optimal replacement females (i.e. females achieving first estrus at an earlier age). A physical characteristic (anogenital distance) and advanced molecular techniques (gene expression) were evaluated for their potential suitability to estimate age at first estrus. In this study, the longer anogenital distance in females that achieved estrus are in line with Seyfang et al. (2018), Mills et al (2020), and Romoser et al. (2020) who report females with longer anogenital distance, greater vulva width, and higher vulva score were associated with greater reproductive success. The specific age at which these measurements were taken varied considerably from 21 days of age (Mills et al., 2020), 15 weeks of age (Romoser et al., 2020), 24 weeks of age (Seyfang et al., 2018) and 10 weeks of age (this study) and each use a different morphometric evaluation. While morphometric tools appear suitable as predictors there is large potential for subjectivity in these measurements. For example, vulva clenching, fecal compaction, and overall gilt restlessness and fidgeting behaviors were routinely exhibited and can impact the morphometric measurement despite efforts to minimize these factors. To effectively retrieve the measurements, allowing the female to relax added considerable amounts of time in sample collection, which would not be practical for on-farm use as a predictor of estrus. More recent evaluation of camera imaging to assess vulva morphometrics may be more suitable (Cruz-Vigo et al., 2022); although images were used to predict day of standing estrus during the estrous cycle and not prior to pubertal attainment in young females. Overall, the use of anogenital distance as a practical on-farm prepubertal marker of reproductive development seems limited.

Within this study, the presence of serum progesterone supports the visual identification of standing estrus and supports the hypothesis that changes in vaginal gene expression in the prepubertal phase can be correlated to reproductive development and standing estrus. Changes in vaginal gene expression were detected and most prominent at 160 days of age indicating suitability of differential vaginal gene expression as a potential tool for estimating age at first estrus. Of particular interest is identifying females likely to achieve estrus at a younger age (i.e. <190 days of age) versus those achieving estrus later (i.e. >200 days of age) or not at all. Understanding the role circulating reproductive hormones play in gene expression is key to differentiating between females that will achieve early or late puberty.

There is limited data on how estrogen impacts TLR-4 production specifically in the vaginal epithelium. Yao et al. (2007) reported that estrogen upregulated TLR-4 expression in the vaginal epithelium of mice during the estrous cycle which is consistent with our results. Clapper et al. (2021) reported that estrogen rises around standing estrus, which is consistent with our own dataset in which ER-α expression was readily detected at standing estrus and would correlate to high estrogen levels. The decrease in ER-α expression at d 104 coincides with the negative feedback action of estrogen that occurs during follicular development (Uenoyama et al., 2021). The IGF-1 expression increased at d160, but the decrease in expression at d104 coincides with Clapper et al. (2001) who reported serum concentrations of IGF-I in gilts decreased from d 84-98 of age. The increase in TAC-3 expression at d160 may be due to the activation of the hypothalamic-pituitary-gonadal axis. The TAC-3 gene possesses the neurokinin-B receptor which is necessary to induce secretion of kisspeptin (Burke et al., 2006). Kisspeptin, in turn, stimulates gonadotropin secretion in prepubertal animals, which eventually causes luteinizing hormone secretion and subsequent ovulation (Lents et al., 2008). In females, this axis becomes activated around puberty, which leads to the onset of reproductive cycling (Thornson et al., 2018).

Overall, this work supports the hypothesis that there are distinct changes in vaginal epithelium based on the increase in IGF-1, TAC-3, TLR-4, and TLR-5 expression between the presumed completion of folliculogenesis (d130) and the start of boar exposure (d160). To definitively determine the potential for prediction of age at puberty, a broader screening of differentially expressed genes specifically comparing larger cohorts of females expressing estrus at ‘early’ versus ‘late’ age and those deemed as ‘anestrus’. The ability to differentiate females considered to have greater reproductive success has the potential for significant savings for swine production systems.

## Conclusion

There are distinct changes gene expression within the vaginal epithelium associated with progression towards first standing estrus. Further investigation into gene expression changes as potential biomarkers of age at standing estrus is warranted.
